# Learning Abilities in a Population of Italian Healthy Preterm Children at the End of Primary School

**DOI:** 10.3390/ijerph17207599

**Published:** 2020-10-19

**Authors:** Silvia Bucci, Francesca Bevilacqua, Chiara De Marchis, Maria Franca Coletti, Simonetta Gentile, Anna Maria Dall’Oglio

**Affiliations:** 1 Unit of Clinical Psychology, Department of Neurological and Psychiatric Sciences, Bambino Gesù Children’s Hospital, IRCCS, 00165 Rome, Italy; mfcoletti@libero.it (M.F.C.); s.gentile6@lumsa.it (S.G.); amdalloglio@libero.it (A.M.D.); 2 Department of Neonatal Medicine and Surgery, Bambino Gesù Children’s Hospital, IRCCS, 00165 Rome, Italy; chiara.demarchis@opbg.net

**Keywords:** very preterm, healthy preterm, learning abilities, neurocognitive profile, clinical neurodevelopment follow-up in preterm children, learning impairment

## Abstract

*Background*: Delays in learning skills have been extensively reported for very preterm children. However, few studies have examined academic achievement profiles in Italian preterm children as a function of their neonatal immaturity. *Methods:* A cross-sectional study was performed that included 82 healthy Italian children born very and extremely preterm (without major neurosensory outcomes; IQ ≥85). Children were evaluated for academic and neurocognitive performances at the second cycle of primary school. *Results*: Healthy preterm children showed on average academic and neurocognitive profiles that did not differ according to gestational age. Impairment was seen to one or more learning domains in 14.6% of the healthy preterm children. Conclusions: Italian children born very and extremely preterm without major neurosensory damage and/or cognitive delay showed on average learning and neurocognitive profiles within the normal range, regardless of gestational age. Nevertheless, they showed higher proportions of learning impairment than a normative Italian population during their final years of primary school. Healthcare providers should be aware of this result, and long-term surveillance should be organized to promptly identify those children who are in need of therapeutic intervention.

## 1. Introduction

Over the last 20 years, there has been an increase in the number of children born preterm, with steadily decreasing gestational age and decreasing neonatal morbidity [1]. Long-term developmental sequelae for children with preterm births are therefore of growing public-health concern [1]. Moreover, cognitive and neuropsychological impairments far surpass the rates of neurosensory disabilities in very preterm children (VP; gestational age, <32 weeks) as well as in those who are extremely preterm (EP; gestational age, <28 weeks) [2]. Even in children who are born preterm with no ‘major’ neurosensory damage (i.e., healthy preterm children), a series of problems is often observed, especially from the age of 5–6 years. These problems involve different neuropsychological processes, including language, visuoperceptive and visual-motor skills, executive functions (e.g., attention) and working memory. These differences from the mean can also be seen for their general IQs [2,3,4,5,6,7,8,9,10,11,12,13,14]. On average, VP children have mean IQs that are within the normal range, although lower than those of typically developing (TD) children [10,11,13,15,16].

These differences in neurocognitive profiles have been generically defined in some studies as “soft signs”, or the typical profile of the VP child [17], and in some meta-analyses, they have been termed “deficits” or “disorders” [1,2,14,18]. It is relevant that these neurocognitive differences might have cascading effects on the later development of these VP children, which might lead to increasingly divergent trajectories for them across multiple domains [13,16,19,20]. In particular, these soft signs can give rise to learning disorders [4,12,21], and specific learning difficulties appear to be present both in children born EP and in part in children born VP, as shown by the well-known meta-analysis of Aarnoudse-Moens [22,23]. This Aarnoudse-Moens study demonstrated that in tests of mathematics, reading, language, verbal fluency, working memory and cognitive flexibility, premature children gained lower mean scores (i.e., between 0.48 and 0.76 standard deviations [SDs]) compared to control children. Brydges et al. [1] confirmed lower mean scores for executive function and processing speed (0.51 and 0.49 SDs, respectively). For school age, as well as in preschool, the delays in academic achievement persisted in VP children also after controlling for general IQs [1,13,14,18,24,25]. Moreover, as suggested in the literature, when investigating academic achievements, it is important to evaluate the neuropsychological domains, including attention, working memory, visual-motor abilities, phonological awareness, fluency and rapid automatized naming [2,12,13,14,22,26,27,28,29]. Menghini et al. reported, for example, that developmental dyslexia is a multifactorial deficit, and that reading is a complex cognitive process that not only involves phonological skills, but also auditory sensory processes, memory abilities, attention processes, automatization, and visual-spatial skills [30]. In particular, a more recent study on a population of Italian children with developmental dyslexia documented that visual-spatial processes have crucial roles in reading skills [31]. These results confirmed that exploring neuropsychological profiles provides better understanding of the characteristics of academic deficits, and better defines the rehabilitative therapeutic implications both at preschool age and once in school [2,12,13,29,32,33].

According to the World Health Organization, 20% of preterm infants have learning disabilities. Some studies have shown that VP infants are 3 to 5 times more likely to experience difficulties in a range of basic school skills and educational domains [6,34]. In particular, higher incidence of difficulties has been shown in the area of mathematics, followed by spelling difficulties [12,35,36]. These difficulties appear to be due to immature development of the central nervous system, which tends to persist throughout the developmental age and up to adulthood [22]. In other words, the developmental profile of preterm children amounts to an atypical profile, rather than the result of a developmental delay [23,26]. In the so-called late preterm children (gestational age, 34 to 36 weeks), instead, these problematic aspects tend to be less evident [37] and to decrease over time, until they are no longer clinically detectable [38]. However, even in adolescence, these children can remain with lower comprehension skills compared to normal text decoding skills [39]. Moreover, to determine the incidence of learning disabilities without bias, it is important to perform the learning assessment in the second cycle of primary school (i.e., after the end of the second year of primary school), when a diagnosis of learning disabilities can be carried out [16,27,40,41,42].

It is also important to differentiate between opaque languages and transparent languages, in terms of orthographic complexity. Opaque languages, such as English, are characterized by a complex relationship between graphemes and phonemes, where each grapheme can correspond to more than one phoneme. Transparent languages, such as Italian, are characterized by direct and predictable relationships between graphemes and phonemes, whereby each grapheme corresponds to a single phoneme [42,43]. Most studies on learning outcomes are on English speaking populations [13,14,16,44]. For an opaque language such as English, there are potentially adverse factors that can influence those predisposed to reading and writing disorders. In contrast, few studies have investigated long-term effects of preterm birth on learning abilities in a transparent language, such as Italian.

To our knowledge, the only follow-up study on long-term learning abilities in an Italian population of premature children was by Guarini and colleagues [26]. Their sample consisted of 37 VP children (gestational age, <32 weeks) at nine years of age, and they reported that 10.8% of these showed a learning deficit. This is in line with other studies in VP children, although with an orthographically irregular language; i.e., an opaque language [12]. The proportion of learning difficulties in the Guarini et al. (2019) study of these Italian VP children was three times that of the Italian population (3.5%) [29]. Interestingly, comorbidities among these learning disabilities in these VP children were proportionally lower than in full-term children with specific learning disabilities, at 2.7% for each category of reading, spelling and mathematics in VP children, vs. 50.1% in those with specific learning disabilities [26]. Among the future prospective of their study, Guarini et al. (2019) advocated studies with larger sample sizes to investigate differences in the academic achievement profiles of VP children as a function of their neonatal immaturity [26].

The present study was aimed at determination of the learning abilities for reading, spelling and mathematics during the second cycle of primary school (8–10 years of age) in Italian preterm children as a function of their neonatal immaturity. A secondary aim was to explore the aspects of neurocognitive abilities, such as visual-motor, attention and short-term memory abilities, and their correlation with the learning profiles.

## 2. Methods

### 2.1. Participants

This is a cross-sectional study that was carried out at the Bambino Gesù Children’s Hospital, a large referral pediatric hospital and clinical research institute in Rome (Italy).

The sample included a cohort of preterm children who were enrolled in the follow-up program of the hospital between May 1997 and February 2007, and who were then evaluated at the age of 8–10 years. The eligibility criteria were: (a) gestational age <33 weeks; (b) absence of congenital malformations; (c) neonatal course free from major neurological and sensory morbidities (e.g., no grade 3–4 intraventricular hemorrhage, according to Papile [45], no cystic periventricular leukomalacia, no seizures, no grade 3–4 retinopathy of prematurity); (d) an IQ ≥85; and (e) Italian monolingual. The sample was divided in two subgroups according to gestational age, as EP (gestational age, <28 weeks) and VP (gestational age, 28–32 weeks) children. The clinical and demographic data of the children and their parents and the data on school support and therapeutic interventions were collected.

In all, 129 monolingual Italian children were consecutively enrolled in the pediatric and neurologic long-term follow-up program of the Bambino Gesù Children’s Hospital. The data for this study were obtained from the medical records collected between January 2008 and June 2015. Forty-seven children (36.4%) were excluded: 21 children because of neurological morbidities (grade 3–4 intraventricular hemorrhage, cystic periventricular leukomalacia, seizures), 12 children because of IQ < 85, 4 children because of grade 3–4 retinopathy, 8 children because they were deaf, and 2 children because they have associated genetic syndrome. No differences were found between included and excluded children in terms of their clinical and demographic data.

Therefore, the study population comprised 82 children: 22 children were born as EP (gestational age, <28 weeks), and 60 children were born as VP (gestational age, 28–32 weeks). The clinical and demographic data of the children and their parents, and the data on the school support and therapeutic interventions, are summarized in Table 1. The mean ages for these groups were (±SD): EP children, 9.43 (±1.26) years; VP children, 9.44 (±0.82) years. There were no significant differences between the groups for age, gender, parent age and educational level (expressed in educational years), school support and therapeutic interventions (Table 1).

### 2.2. Procedures

The assessment of the children was performed in a quiet room at the Hospital in the Clinical Psychology Unit, by a trained psychologist.

The study protocol met the ethical guidelines for the protection of human participants and received formal approval from the local Ethics Committee (IRCCS Paediatric Hospital Bambino Gesù). The parents of the children gave their written informed consent for participation in the study.

The tests used in this study are given in Table 2, with further details in Appendix A.

### 2.3. Statistical Analysis

All of the statistical analyses were carried out using SPSS, version 20, for Windows. The significance levels for all of the tests was set to *p* < 0.05. Before analyzing the differences in learning abilities and neurocognitive profiles, we evaluated whether the preterm groups differed in their sociodemographic characteristics, such as age, gender and the educational level of both parents. Chi-squared tests were used for discrete variables (gender) and ANOVA for continuous variables (age, parent educational level [educational years]). Most of tests used do not provide standardized scores, and the means and standard deviation of these tests changed according to the class and/or age of the child. To make the scores comparable, they were z-standardized (M = 0, SD = 1) according to normative sample mean and SD values. The raw values obtained in the preterm group were transformed into z-scores by computing the distance between each individual value and the reference mean, divided by the SD: z = (observed score value − reference mean value)/SD (raw scores of the variables are given in Appendix B). To verify the normality of the distributions of the variables taken into consideration in the study, the averages, standard deviations and indices of skewness (asymmetry) and kurtosis (kurtosis) were examined. The scales have values of skewness and kurtosis close to or lower than |1|. In addition, The Kolmogorov–Smirnov test was used to check for violations of the assumption of normal distributions. Three variables (i.e., test accuracy, word accuracy, no word accuracy) showed significant values to the Kolmogorov–Smirnov test, attesting to the abnormality of the distribution of data. Differences in learning abilities and in neurocognitive profiles between the preterm (EP/VP) and normative data, were evaluated by ANOVA. For each score, differences between the sub-groups were analyzed using Tukey HSD (“honestly significant difference”) post-hoc tests. Pearson correlations were performed to investigate the interrelationships between learning ability and neuropsychological profiles. For the profiles of the learning difficulties, the mean z-scores were calculated for each learning area (reading: word, non-word, text speed, accuracy; spelling: word, non-word; mathematics: four domains in AC-MT, calculus quotient for the Battery for Developmental Dyscalculia [BDE] test). These z-scores were classified as: impaired (≤−2); at risk (−2 to −1), and average range (≥−1). The impaired learning areas (≤−2) were further classified as isolated or in comorbidity.

## 3. Results

### 3.1. Learning Abilities

The mean z-scores for the EP and VP children fell within the normal range in all of the learning tests.

Reading. The EP and VP children showed no differences in terms of speed, accuracy and comprehension. The EP children scored significantly lower than the normative sample in a reading task of non-words (non-word speed, F = 4.45; *p* = 0.012).

Spelling. No significant differences emerged in the spelling tasks (word and non-word dictation) between the EP and VP. EP children scored significantly lower than the normative sample in a dictation task of words (F = 9.93; *p* = 0.000).

Mathematics. The EP and VP children showed no differences in mathematical skills. The EP children showed only a trend to lower scores than the VP children, although the EP children achieved significantly lower scores than the normative sample (AC-MT written calculation, F = 8.79; *p* = 0.000; AC-MT accuracy, F = 4.40, *p* = 0.012; Battery for Developmental Dyscalculia, F = 6.97, *p* = 0.001).

**Figure 1:**
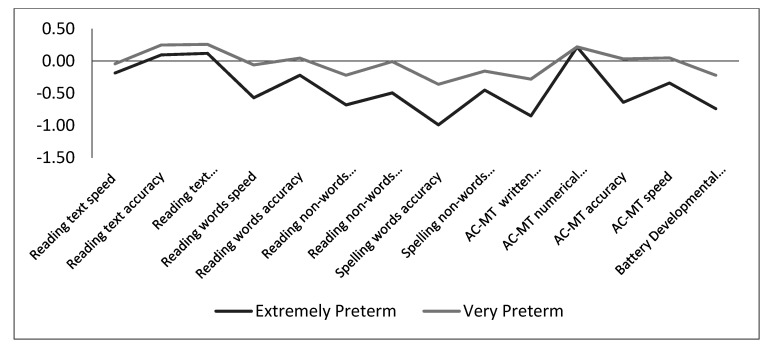
Learning profiles for the extremely preterm and very preterm children.

The z scores for all learning tests of the EP and VP children are given in Table 3, and the comparison of the group profiles is shown in Figure 1. The raw scores for all learning tests are reported in Table A1, Table A2 and Table A3 in Appendix B.

### 3.2. Neurocognitive Abilities

IQ. The mean IQs of the EP and VP children did not differ significantly (Table 4).

The mean z-scores for the EP and VP children fell within the normal range for children for all of the neurocognitive tests.

Spatial abilities. No significant differences emerged between the EP and VP children and with the normative sample in any of the three subtests.

Attention and visual processing speed. No significant differences emerged between the EP and VP children. The EP children showed lower score than the normative sample for sustained attention (F = 6.53, *p* = 0.002).

Short-term memory. No significant differences emerged in the verbal (Digit test) and visuospatial sequential (Corsi test) short-term memory tests between the EP and VP children.

**Figure 2:**
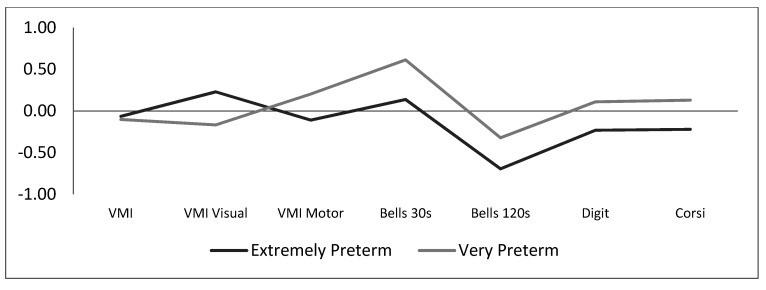
Neurocognitive profiles for the extremely preterm and very preterm children.

The z scores of the EP and VP children for all neurocognitive tests are given in Table 4, and the comparisons of the group profiles are shown in Figure 2. The scores for all neurocognitive tests are reported in Table A4 in Appendix B.

### 3.3. Correlations between Learning Abilities and Neurocognitive Profiles

The reading skills were correlated with verbal short-term memory, visual processing skills and motor component of the visual-motor skills. The writing skills were correlated with verbal short-term memory and attention. The mathematics skills were correlated with verbal and visuospatial short-term memory. All of these correlations are given in Table 5.

### 3.4. Learning Profiles and Comorbidities

Table 2: at risk (−2 to −1) or in the average range (≥−1) are summarized in Table 6. Overall, 12 of the children (14.6%) had impairment in one or more of the learning domains, and half of the children had one or more in the risk domain. The prevalence of these learning impairments and comorbidities are summarized in Table 7.

## 4. Discussion

The primary goal here was to compare Italian children born highly preterm but without major neurosensory outcomes and with IQ ≥ 85 as a function of their neonatal immaturity, in terms of their academic and neurocognitive performances at the second cycle of primary school, at 8–10 years of age.

### 4.1. Learning Abilities

On average, the preterm children showed academic performances that were within the normal range regardless of their gestational age, although there was a trend towards the EP children being less competent than the VP children in terms of mathematical skills. Our results are in line with those of Saavalainen et al. [46], who observed that their extremely preterm group with a gestational age of 29 weeks did not differ from the more mature group with a gestational age between 30 and 32 weeks, in terms of their school grade points. Both of these studies considered populations with transparent languages (i.e., Italian and Finnish). It is worth noting that the EP children of our sample showed performances in the lower part of the normal range, which reached statistically significant differences from the normative mean only for non-words reading speed, word spelling and mathematical skills. When interpreting the data, it must be kept in mind that we compared scores obtained by the EP and VP children with normative data of tests, and not with scores of the TD children (control group). It is possible that this influenced our results, considering that several meta-analyses and reviews of studies with control groups have reported more significant differences [1,14,22].

In a recent longitudinal study, Twilhaar et al. [47] reported significant differences between TD children controls and VP children, who scored 0.53 SD lower for mathematics, 0.31 SD lower for reading comprehension, and 0.21 SD lower for spelling. In other words, they clearly indicated that the differences between the VP and TD children were well below one SD. They also highlighted how these difficulties and differences persisted throughout primary school. It is important to note that they defined difficulties starting from a difference of 0.50 SD from their controls.

When interpreting such data, we should consider that Italian Law on learning disabilities defines the diagnosis of specific learning disabilities as a difference of ≥2 SD from the norm in one or more academic skills. Thereafter, the mean academic scores of the children in the present study would not fall into a category of learning disabilities, but only of ‘at risk’ or with minor difficulties. In other words, this would not be sufficient to certify a specific learning disorder according to Italian law (Lg. 170) [27,28,32,42,48,49,50,51]. This is in line with what has been reported in other studies and meta-analyses that have analyzed preterm profiles within the normal range, where they have nevertheless shown significant differences compared to full-term children in different areas of learning or in neurocognitive domains [2,14,18,22,47,52]. As clinicians, we wonder about the clinical meaning of these differences in the everyday life of a child, and we believe that it is not easy to decide if and when to recommend rehabilitation.

### 4.2. Cognitive Profile and Neurocognitive Abilities

The IQs of the EP and VP children fell within the normal range, as required by the inclusion criteria. Indeed, it is important to note that we not only excluded children with intellectual deficits (IQ < 70) but also those falling into the borderline range of IQ (i.e., IQ 70 to 84). In line with other studies, we chose to do this to prevent bias in the identification of specific learning disabilities that are not influenced by intellectual impairment [21]. We did not find any significant differences between the IQ scores of the EP and VP children. The literature has reported that lower gestational age is correlated with higher risk of acute events, such as asphyxia and/or cerebral palsy, which can result in serious damage to the central nervous system and long-lasting developmental outcomes [3,53]. We can speculate that should these preterm children remain ‘unscathed’ by such acute events in the perinatal period, these so-called ‘healthy preterm children’ will then show similar developmental trajectories regardless of gestational age [47,52,54]. This speculation is also confirmed by our results that show similar proportions of EP and VP children who needed school support and/or therapeutic interventions.

When considering the neurocognitive domains (visual motor, attention and visual processing, and short verbal and sequential spatial short memory), the profiles were similar to the cognitive development. So, the EP and VP children showed performances within the normal range. This is in agreement with Sansavini et al. [13], who reported that when considering a wide range of gestational ages and excluding children with severe cerebral damage and heavy socio-economic disadvantage, the scores of preterm children might lie within the normal range, even if they remain lower than full-term controls [13]. Moreover, some studies have reported a catching-up of some basic competencies (e.g., receptive lexicon, verbal short-term memory) from preschool and school age to adolescence, with long-lasting effects of preterm birth on more complex competencies (e.g., complex linguistic functions, mathematics, executive functions) [13].

Working memory is one of the crucial executive functions for meeting the challenges of daily life and performing academic tasks. A recent study used functional magnetic resonance imaging to better understand and define differences in the visuospatial working memory network of school-aged preterm children [55]. This study reported that younger preterm children were low-performing and showed only a small frontal activation cluster. Older preterm children were high-performing and showed activation that was similar to the working memory network of the total group of term-born controls. They hypothesized that younger, low-performing, VP children cannot engage the same widespread network seen in their control children, because they are still in the process of functional organization of their working memory, which leads to different network characteristics. The delayed ongoing maturation of the frontal lobes as a consequence of the premature birth might be one reason for alterations in their neurofunctional development [55]. This study also stressed that their sample was composed of relatively healthy preterm children, and that this might have contributed to catch-up of their functional development, rather than persistent functional alterations. This hypothesis can also explain our data, where there were performances within normal range as to short-term visuospatial and verbal memory in both the EP and VP children.

The present study used some domains for executive functions, visuospatial processing and sensory-motor skills to explore correlations between the neurocognitive abilities and learning skills, and to thus to orient potential targets for intervention.

### 4.3. Correlations between Learning Abilities and Neurocognitive Abilities

The data from the correlation analysis in the present study confirm that the neurocognitive domains explored are related to the learning abilities. The analysis performed allows only covariations to be appreciated, and not causal relationships. However, as reported by Menghini et al., casual relationships between neurocognitive domains and learning abilities are particularly difficult to assess [56]. As summarized by Sansavini et al. [13], the characteristics and extents of deficits vary as a function of the complex interactions among biological and environmental constraints, developmental timing and type of competence, which highlights the dynamic process of development.

Nonetheless, neurocognitive profiles give us important information with respect to academic skills. In particular, in our sample, mathematics showed correlations with verbal and visuospatial short-term memory, which is in line with the literature [2,14,55]. For reading skills (i.e., accuracy, text comprehension), these were correlated to verbal short-term memory and visual-motor skills. When considering writing skills, these correlated with verbal short-term memory, and attention. These correlations confirm the evidence from studies on both transparent [13,16] and opaque [1,2,18,22] languages. They also confirm the usefulness of exploring the neurocognitive domains, and in particular, those that are inherent to the executive functions, to identify therapeutic and preventive interventions from preschool age [11,57,58]. For EP children relative to term-born controls from school entry through adolescence, Johnson et al. [2] reported significant deficits in a range of basic cognitive processes, which included short-term memory, processing speed, visual-perceptual skills, sensorimotor integration and attention [2]. Even without neurosensory impairment, EP children are at high risk for multiple intellectual and learning disabilities that can impact on their school performance [12]. In addition, those without significant learning disabilities can have poor neuropsychological skills that impact on their performance at school. Problems in multiple neurocognitive domains can increasingly limit children’s learning opportunities, with cascading effects on development over time. Those findings have shown how improving executive functions and visuospatial skills following an EP birth represents an important target for intervention [12].

It is worth noting that among the neurocognitive domains, only visual-motor skills were correlated to IQ. This suggests the importance of long-term neurocognitive follow-up even if the children have an IQ within the normal range [1,11,13,58,59,60,61]. Nevertheless, in agreement with the literature, the IQs were correlated with text comprehension [12,13,27,32,48,62].

When considering gestational age, this showed positive correlation only with visuospatial short-term memory. In other words, the shorter the gestational age, the worst the performances in this domain. We did not find significant correlations with other neurocognitive domains. This finding is in contrast to other studies that have reported correlation with the motor component of visual-motor skills, which thus suggested persistent micro-motor damage in preterm children that can lead to dysgraphia and spelling difficulties [13,63]. Moreover, a meta-analysis on motor development in VP children from infancy to adolescence suggested that they were an average of −0.57 to −0.88 SD behind their full-term peers [13,64].

When analyzing the correlations between gestational age and learning abilities, we did not find any significant correlations that are in contrast with previous studies that have described correlations with mathematics [18,65] and with reading speed and text comprehension [16].

Finally here, all of these learning tests correlated with each other, which confirms the high risk of comorbidity between various learning domains [12,20,26]. In particular, it is interesting to note that the mathematics tests were correlated with text comprehension, which confirms the relationship between reading comprehension skills and mathematics, especially for the aspects of understanding the elements necessary to perform a problem-solving task [13,18,32,66].

### 4.4. Proportion of Impaired Children

Overall, in this sample of healthy preterm children, 12 of them (14.6%) showed one or more learning domains that were impaired (≥2 SD under the norm), and half of them were also rated as risk performances in one or more domains (1–2 SD under the norm). These data are in line with Guarini et al. [16] in VP children screened at the end of the second year of primary school. However, in our study we also considered the gestational age of children preterm, and we found that the incidence rate of learning disabilities was greater in the EP children (reading, 13.6%; spelling, 10.5%; mathematics, 19.0%) than in the VP children (reading, 1.7%; spelling, 5.9%; mathematics, 5.7%). The present study confirms that these difficulties also persist during the last years of primary school [13,22,52]. As previously reported, the World Health Organisation has indicated higher proportions of impairment in preterm children (20%), as have other studies that have considered preterm children in countries with opaque languages (25–40%, [67]; 30–50%, [68]; >40%, [69]. We can speculate that this difference from the present study is influenced by two factors: the present study included only healthy preterm children, and Italian is a transparent language.

Furthermore, in the present study, it emerged that 18.3% of the children showed performances at risk in one or more of the learning domains, with higher proportions for the EP children (EP: reading, 9.1%; spelling, 26.3%; mathematics 9.5%; VP: reading, 6.7%; spelling, 11.8%; mathematics, 7.5%). On the whole, almost half of the preterm children here underwent therapeutic interventions (e.g., speech therapy and/or psycho-motor rehabilitation), and almost 30% of them had specific school support (i.e., special teacher and/or educator). There were no differences between the EP and VP children, even though the proportion of learning impairment of EP were higher than those of the VP children. This result demonstrates the social impact of this population.

Comparisons with the proportions of learning impairments of the Italian population are hampered by methodological differences, mostly concerning the diagnostic criteria, but also the definitions of impairment and risk. Barbiero et al. [29] reported that studies conducted in Italy are limited and affected by this variability, with a prevalence of learning impairment that ranges from 1.3% to 8.5%. National guidelines have reported prevalence that ranges from 2% to 3.5% [27]. The present study appears to confirm that a child born VP is 2 to 5 times more likely to have difficulties across a range of basic school skills and educational domains [6,34]. In our opinion, this is particularly important to know, considering that we excluded children with cognitive impairment and with major neurological and sensory morbidities. In other words, even if a child does not suffer from major neurosensory damage and/or cognitive delay, if born preterm, the child maintains a significantly higher risk for development of a learning disability. In this regard, it is important to know that some studies have differentiated the learning-impaired profiles of preterm children from those of full-term children with a diagnosis of a specific learning disorder. Guarini et al. [26] stated that the atypical academic achievement profiles in VP children cannot be considered to be similar to those of children with specific learning disorders, as learning delays were less widespread, and involved only some learning abilities, and were less severe. The profile analysis showed that preterm birth increases the risk of learning delays, but not of disorders, bringing further and more generalized evidence to the hypothesis of Jaekel and Wolke [65] for mathematics difficulties. They suggested that preterm birth does not increase the risk of dyscalculia, but lower gestational age is associated with higher risk of mathematic impairment.

It is not easy to compare studies, as they often refer to assessments performed with different tools, at different ages, and with different interpretation criteria. The diagnosis of Specific Learning Disabilities cannot be formulated in the first stages of reading and writing acquisition, as enough time needs to be allowed for the teaching and learning processes to be completed. Thus, dyslexia and dysorthography are typically diagnosed from the end of the second grade, and dyscalculia from the end of the third grade [42]. A strength of our study is thus the period of assessment (years 3–5 of primary school), which minimizes the risk of false positives. For the interpretation criteria, the diagnostic manual ICD-10 indicates a cut-off of 2 SD (or the fifth percentile) below the mean to identify poor performance in academic tests. However, in the literature on dyslexia, cut-off levels are often much higher (25th or 15th percentiles) [42]. Recent studies have suggested that only children whose performance falls below the 10th percentile in at least two specific tests of basic arithmetic skills should be considered as dyscalculic [42]. In Italy, the law that governs the needs and rights of children with Specific Learning Disabilities is relatively recent [27,28], and there is still wide debate on this that involves both the law and clinical results [32,48,49,59]. That is why we chose to consider two classes of severity in the present study (i.e., impaired, at risk) to describe the learning outcomes of these preterm children. We believe that children in both classes of severity experience discomfort in their school life, which must be taken into consideration. This discomfort can cause cascading effects, on both learning and behavior, and also on the self-perception of the child [70]. On this basis, it is important to also consider comorbidities with behavioral problems [12], as well as the social and emotional development of the children, which we have not evaluated in the present study.

The present study has several limitations that should be addressed. First, the number of children included was limited and the study did not include a control group of TD children. Secondly, during the neuropsychological follow-up, for the evaluation of mathematical skills, the AC-MT was replaced by the BDE, which was considered as a more suitable tool for the follow-up setting. This led to some of the children being evaluated with the AC-MT scale, and some of them with the BDE scale, so that it is not possible to compare the means over all of these children. Thirdly, the preterm children included were born with a gestation age <33 weeks, although the World Health Organisation defines VP births as those before the completion of 32 weeks of gestation. On the one hand, this partially limits the possibility to compare the present study with other studies, but on the other hand, it respects our population of children who are enrolled for hospital neurodevelopment follow-up [11], and many other studies have used the same criteria [69,71]. Fourthly, although several competences were evaluated in the present study to better understand the relationships between neurocognitive and learning skills, other important executive functions should be considered in further studies. Moreover, the analysis performed allows only covariations to be appreciated, and not causal relationships. Finally, even in the presence of acceptable skewness and kurtosis, three of the variables considered in the present study (i.e., test accuracy, word accuracy, no word accuracy) showed significant values to the Kolmogorov–Smirnov test, attesting to the abnormality of the distribution of data. This should make us cautious in the interpretation of research results.

## 5. Conclusions

When preterm children are not affected by acute events associated with major neurosensory damage and/or cognitive delay in their perinatal period, they show similar academic and neurocognitive trajectories regardless of gestational age. Moreover, although the learning profiles of the preterm children fell within normal range, these so-called “healthy preterm children” show higher levels of learning impairment during their last years of primary school than the normative Italian population. Healthcare providers should be aware of this result, and long-term surveillance should be organized to promptly identify those children who are in need of therapeutic intervention.

## Figures and Tables

**Table 1:** Clinical characteristics of the preterm children enrolled in the study.

Clinical Characteristic	Extremely Preterm	Very Preterm	*p*
	N = 22	N = 60	
Gender (males) [n (%)]	11 (50.0)	36 (60.0)	NS
Gestational age (week) (mean ± SD)	26.4 ± 1.06	30.3 ± 1.36	0.00
Birth weight (g) (mean ± SD)	955.4 ± 208.8	1387 ± 371.87	0.00
Age (mean ± SD)	9.43 ± 1.26	9.44 ± 0.82	NS
Mother’s education level (mean ± SD)	13.71±3.01	13.47 ± 3.81	NS
Father’s education level (mean ± SD)	13.24 ± 4.06	12.7 ± 3.76	NS
Mother’s Age (mean ± SD)	41.3±6.1	40.2 ± 7.4	NS
Father’s Age (mean ± SD)	44.2±5.2	43.8 ± 4.4	NS
School support [n (%)]	6 (27.3)	18 (30.0)	NS
Therapeutic interventions [n (%)]	10 (45.5)	27 (45.0)	NS

**Table 2:** Tests used at the follow-up (aged 8–10 years).

Target	Test	Reference
**Learning abilities**		
Reading (speed, accuracy, comprehension)	Cornoldi Memory and Training (MT)	Cornoldi and Colpo, 2010
	Battery for Dyslexia and Developmental Dysorthography	Sartori et al., 1995
Writing (dictation of words and non-words)	Battery for Dyslexia and Developmental Dysorthography	Sartori et al., 1995
Maths	Cornoldi Calculation Ability (AC-MT)	Cornoldi et al., 2002
	Battery for Developmental Dyscalculia (BDE)	Biancardi and Nicoletti, 2004
**Cognitive development**	Wechsler Intelligence Scale for Children (WISC) III	Weschsler, 2006
	Raven’s Coloured Progressive Matrices (CPM)	Raven, J. C., 2006
**Neurocognitive abilities**		
Spatial abilities	Visual-Motor Integration Test	Beery et al., 2000
Attention and visual processing	Bell Test	Biancardi, 1997
Short-term memory	Verbal—Digit	Orsini et al., 1987
	Spatial—Corsi’s Block Tapping	Orsini et al., 1987

**Table 3:** Z-test scores for the learning skills of the extremely preterm and very preterm children.

Learning Skill	Sub-Skill	Z-Test Score (Mean ±SD)			
		Extremely Preterm	Very Preterm	Normative Sample	*p*
Reading	Text speed	−0.18 ± 0.92	−0.04 ± 0.86	0.00 ± 1.00	0.66
	Text accuracy	0.09 ± 0.75	0.25 ± 0.67	0.00 ± 1.00	0.14
	Text comprehension	0.12 ± 0.73	0.26 ± 0.68	0.00 ± 1.00	0.12
	Word speed	−0.57 ± 2.40	−0.06 ± 0.98	0.00 ± 1.00	0.08
	Word accuracy	−0.22 ± 1.33	0.046 ± 0.87	0.00 ± 1.00	0.55
	Non-word speed	−0.68 ± 2.08	−0.22 ± 0.96	0.00 ± 1.00	0.01
	Non-word accuracy	−0.49 ± 1.73	−0.07 ± 0.98	0.00 ± 1.00	0.11
Spelling	Word accuracy	−0.99 ± 1.66	−0.36 ± 1.21	0.00 ± 1.00	0.00
	Non-word accuracy	−0.45 ± 1.41	−0.15 ± 1.06	0.00 ± 1.00	0.10
Mathematics	AC-MT written calculation	−0.85 ± 1.60	−0.28 ± 1.09	0.00 ± 1.00	0.00
	AC-MT numerical knowledge	0.22 ± 0.61	0.22 ± 0.59	0.00 ± 1.00	0.14
	AC-MT accuracy	−0.64 ± 1.06	0.03 ± 1.06	0.00 ± 1.00	0.01
	AC-MT speed	−0.34 ± 1.43	0.05 ± 0.81	0.00 ± 1.00	0.55
	Battery for Developmental Dyscalculia	−0.74 ± 0.97	−0.22 ± 0.89	0.00 ± 1.00	0.00

*p*: from ANOVA analysis.

**Table 4:** Summary of the neurocognitive skills for the extremely preterm and very preterm children.

Neurocognitive Skill	Sub-Skill	Z-Test Score (Mean ± SD)			
		Extremely Preterm	Very Preterm	Normative SAMPLE	*p*
Intelligence quotient (IQ)	-	101.8 ±11.05	100.1 ± 11.69	100.00 ± 15.00	
Visual-motor integration	Total	−0.06 ±0.72	−0.10 ± 0.88	0.00 ± 1.00	0.78
	Visual processing	0.23 ±1.17	−0.17 ± 1.04	0.00 ± 1.00	0.42
	Motor coordination	−0.11 ±0.85	0.20 ± 1.05	0.00 ± 1.00	0.33
Attention and visual processing	Selective	0.14 ±1.61	0.61 ± 1.43	0.00 ± 1.00	0.01
	Sustained	−0.69 ±0.88	−0.32 ± 0.62	0.00 ± 1.00	0.00
Short-term memory	Verbal	−0.23 ±0.81	−0.11 ± 0.80	0.00 ± 1.00	0.40
	Visuospatial	−0.22 ±0.92	0.13 ± 1.14	0.00 ± 1.00	0.40

*p*: from ANOVA analysis.

**Table 5:** Correlations between the learning abilities and the neurocognitive profiles.

	1	2	3	4	5	6	7	8	9	10	11	12	13	14	15	16	17	18	19	20	21	22
**1. Gestation age**	---																					
**2. IQ**	−0.06	---																				
**3. Reading text speed**	0.04	−0.09	---																			
**4. Reading text accuracy**	0	0.13	0.30 **	---																		
**5. Reading text comprehension**	0.04	0.28 *	0.29 *	0.30 **	---																	
**6. Reading words speed**	−0.04	−0.22	0.66 **	0.45 **	0.2	---																
**7. Reading words accuracy**	−0.01	−0.09	0.34 **	0.45 **	0.13	0.67 **	---															
**8. Reading non-words speed**	−0.02	−0.30 *	0.59 **	0.43 **	0.14	0.88 **	0.57 **	---														
**9. Reading non-words accuracy**	0.03	0.12	0.26 *	0.38 **	0.19	0.62 **	0.76 **	0.53 **	---													
**10. Spelling words accuracy**	0.08	−0.01	0.32 **	0.36 **	0.18	0.47 **	0.49 **	0.38 **	0.42 **	---												
**11. Spelling non-words accuracy**	−0.02	0.07	0.27 *	0.30 *	0.14	0.57 **	0.44 **	0.54 **	0.52 **	0.51 **	---											
**12. AC-MT written calculation**	0.04	0.22	−0.18	0.14	0.06	−0.2	0.05	−0.29	0.23	0.37	0.11	---										
**13. AC-MT number knowledge**	−0.31	0.24	0.03	0.32	0.1	0.14	0.41 *	0.04	0.41	0.52 *	0.02	0.45 *	---									
**14. AC-MT accuracy**	−0.22	0.22	−0.06	0.53 **	0.06	0.31	0.44 *	0.33	0.52 *	0.52 *	0.42 *	0.28	0.69 **	---								
**15. AC-MT speed**	−0.02	0.23	−0.21	0.26	−0.11	0.02	−0.02	−0.03	0.45 *	0.24	0.2	0.49 *	0.36	0.42 *	---							
**16. BDE**	0.2	0.13	0.39 **	0.24	0.43 **	0.46 **	0.43 **	0.48 **	0.51 **	0.49 **	0.24	−-	−-	−-	−-	---						
**17. Visual-motor integration tot**	0.04	0.25 *	0.02	0.37 **	0.13	0.11	0.19	−0.02	0.22	0.12	0.16	0.2	0.05	0.17	0.12	0.01	---					
**18. Visual processing skills**	−0.15	0.26 *	0.09	0.19	0.19	0.09	0.09	0.07	0.2	0.13	0.06	0	0.07	0.13	−0.25	0.2	0.44 **	---				
**19. Motor coordination**	0.16	0.36 **	−0.07	0.13	0.03	−0.01	0.1	−0.06	0.30 *	0.18	0.22	0.31	0.08	0.25	0.17	0.09	0.30 *	0.34 **	---			
**20. Selective attention**	0	0.22	0.08	0.24	0.07	0.07	0.03	0.12	−0.02	0.30 *	0.25	0.19	0.19	0.27	0.24	0.07	−0.30 *	0.11	0.22	---		
**21. Sustained attention**	0.21	0.14	0.07	−0.05	0	0.01	0.03	−0.02	0.01	0.19	0.06	0.22	0.05	0.09	0.15	0.09	0.12	−0.07	0.25	0.46 **	---	
**22. Visuospatial short-term memory**	0.24 *	−0.05	0.02	−0.03	−0.05	−0.01	0.07	−0.05	0.21	0.03	−0.08	0.46 *	0.48 *	0.17	0.40 *	0.25	0.1	0.18	0.07	−0.24	−0.08	---
**23. Verbal short-term memory**	0.06	0.16	0.26 *	0.29 **	0.23 *	0.14	0.01	0.1	0.15	0.32 **	0.16	0.37	0.47 *	0.36	0.47 *	0.35 *	0.18	0.18	0.07	0.08	0.03	0.31 **

*. *p* < 0.05; ** *p* <0.01.

**Table 6:** Summary of the children in each group as impaired (≤−2 SD), at risk (between −2 and −1 SD) or in the average range (≥−1 SD) for the reading, spelling and mathematics skills.

Skill	Children at Risk [n (%)]					
	Extremely Preterm			Very Preterm		
	Impaired	At Risk	Average	Impaired	At Risk	Average
Reading	3 (13.6)	2 (9.1)	17 (77.3)	1 (1.7)	4 (6.7)	55 (91.7)
Spelling	2 (10.5)	5 (26.3)	12 (63.2)	3 (5.9)	6 (11.8)	42 (82.4)
Mathematics	4 (19.0)	2 (9.5)	15 (71.4)	3 (5.7)	4 (7.5)	46 (86.8)

**Table 7:** Summary of the prevalence of the learning impairments and comorbidities in each of the groups of children (z-score cut-off, ≤−2).

Learning Impairment	Children with Impairment [(n (%)]		
	Extremely Preterm	Very Preterm	Total
No deficit	17 (77.3)	53 (88.3)	70 (85.4)
Reading only	0 (0)	1 (1.7)	1 (1.2)
Spelling only	1 (4.5)	4 (6.7)	5 (6.1)
Mathematics only	1 (4.5)	2 (3.3)	3 (3.7)
Reading and Spelling	0 (0)	0 (0)	0 (0)
Reading, Mathematics	2 (9.1)	0 (0)	2 (2.4)
Spelling, Mathematics	0 (0)	0 (0)	0 (0)
Reading, Spelling, Mathematics	1 (4.5)	0 (0)	1 (1.2)
Total Learning deficit	5 (22.7)	7 (11.7)	12 (14.6)

## Appendix A

### Appendix A.1. Learning Abilities

#### Appendix A.1.1. Reading

##### Battery for Dyslexia and Developmental Dysorthography

Sartori et al., 1995. G. Sartori, R. Job and P.E. Tressoldi, Batteria per la valutazione della dislessia e della disortografia evolutiva, Giunti O.S. Organizzazioni Speciali, Firenze (1995).

The Battery for Dyslexia and Developmental Dysorthography (*Batteria per la Dislessia e la Disortografia Evolutiva*; Sartori G 1995) was used here to assess reading speed and accuracy of reading of the children. Two subtests were chosen. The first was for reading speed, and consisted of reading aloud four lists of 28 concrete and abstract words that varied in frequency and concreteness. It started with a list of very common and concrete words, followed by lists of words decreasing in frequency and concreteness (lengths from four to eight letters). The reading speed was calculated by dividing the number of syllables read by the time taken to read them (in seconds). The accuracy was defined by the number of words read incorrectly. In the second subtest, the children were asked to read three lists of 16 orthographic strings aloud (non-word length from five to nine letters), as accurately and rapidly as possible. Again, the reading speed was calculated by dividing the number of syllables read by the time taken to read them (in seconds). The accuracy was defined by the number of non-words read incorrectly. Testing time: minimum 10 min.

##### Cornoldi MT Battery

Cornoldi C. and Colpo G. (2004), Prove di Lettura MT per la Scuola Primaria, Giunti Organizzazioni Speciali, Firenze.

Speed, accuracy and text comprehension were also assessed using the Cornoldi MT Battery (Cornoldi, Stroke, MT Group, 1981). The children read two different paragraphs according to the class they were in. The first one included a read-aloud within a predetermined amount of time, to measure the speed (seconds per syllable) and accuracy (number of errors). The second paragraph was read in silence and without time constraints, and the child then answered multiple-choice questions that enabled the examiner to determine the understanding of the text. Testing time: minimum 10 min.

#### Appendix A.1.2. Writing

##### Battery for Dyslexia and Developmental Dysorthography

Sartori et al., 1995 G. Sartori, R. Job and P.E. Tressoldi, Batteria per la valutazione della dislessia e della disortografia evolutiva, Giunti O.S. Organizzazioni Speciali, Firenze (1995).

The Battery for Dyslexia and Developmental Dysorthography (*Batteria per la Dislessia e la Disortografia Evolutiva*; Sartori G 1995) was also used for evaluation of the writing skills of the children through dictation of words and non-words. Testing time: minimum 10 min.

#### Appendix A.1.3. Mathematics

##### AC-MT Test

Cornoldi C., Lucangeli D. and Bellina M. (2002), Test AC-MT 6–11, Erickson, Trento.

The arithmetic abilities of the children were assessed using the AC-MT test (Cornoldi C. 2002), which enables evaluation of computational and problem-solving skills in schools and clinical settings. The AC-MT test indices were defined as follows: (a) written calculation; (b) numerical knowledge; (c) accuracy; and (d) speed. Testing time: minimum 20 min.

##### BDE Test

Battery for Developmental Dyscalculia (Biancardi and Nicoletti, 2004).

The BDE is an individually administered test that was designed to assess the numerical and arithmetic skills of children in the second cycle of primary school and in sixth grade. The BDE was divided into tests that assessed the numerical and calculation skills. In this case, only the tests relative to calculating skills were administered. The BDE allowed the assignment of a calculation quotient. Testing time: minimum 20 min.

### Appendix A.2. Cognitive Area

#### Appendix A.2.1. Wechsler Intelligence Scale for Children III

Weschsler D. (2006), WISC-III Wechsler Intelligence Scale for Children III, Giunti Organizzazioni Speciali, Firenze.

Wechsler D. (2006), WISC III Wechsler Intelligence Scale for Children-III: contributo alla taratura italiana a cura di A. Orsini e L. Picone, Giunti Organizzazioni Speciali, Firenze.

The WISC-III (Wechsler, 1991) is an individually administered test of intellectual ability for children aged 6 years to 16 years 11 months. It consisted of 10 mandatory and three optional subtests (M = 10, SD = 3) that combined to yield the verbal (VIQ), performance (PIQ) and full scale (FSIQ) IQs (M = 100, SD = 15). The WISC-III also provided factor-based index scores for verbal comprehension, perceptual organization, freedom from distractibility, and processing speed. Extensive evidence of reliability and validity is presented in the WISC–III manual (Wechsler, 1991). Testing time: 90–120 min.

#### Appendix A.2.2. Raven’s Colored Progressive Matrices

Raven J C. (2006), CPM Colored Progressive Matrices, Giunti Organizzazioni Speciali, Firenze.

The CPM is a non-verbal intelligence test that is representative of general intellectual capacity, or the “g” factor proposed by Spearman. The CPM was developed to assess children aged from 5 to 11 years old, mentally disabled individuals, and the elderly. Testing time: minimum 15 min.

### Appendix A.3. Neurocognitive Area

#### Appendix A.3.1. Spatial Abilities

##### Visual-Motor Integration Test

Beery K.E. and Buktenika N.A. (2000) VMI. Developmental Test of Visual-Motor Integration. Il Beery-Buktenika con i test supplementari di percezione visiva e coordinazione motoria. Curatore edizione italiana: Cristina Preda.

The VMI is a ‘paper and pencil’ test that was used for the evaluation of spatial abilities. The children were asked to copy a series of 24 geometric shapes of increasing complexity into a pre-defined space. The accuracy of the copies indicated the score. Two supplemental tests of the VMI were used to assess visual and motor skills separately: (1) visual perception test, which assessed the visual skills of the children by limiting the motor responses to simple pointing (the children matched geometric forms to ones in a given stimulus); and (2) motor coordination test, which helped to determine the level of the fine-motor skills of the children (they traced shapes while remaining inside a double-lined path). The second of these cannot completely eliminate the visual component, but the visual demands were lessened by the presence of strong visual guides for the required motor performance. Total testing time: 10 min.

#### Appendix A.3.2. Attention and Visual Processing

##### Bell Test

Gauthier L, Dehaut F, Joanette Y. The bells test: a quantitative and qualitative test for visual neglect. Int J Clin Neuropsychol 1989; 11: 49–54.

Biancardi A. (1997), Il test delle campanelle modificato: una popolazione per lo studio dell’attenzione in età evolutiva, «Psichiatria dell’infanzia e dell’adolescenza», vol. 64, pp. 73–78.

The Bell test (Biancardi, 1997) enabled evaluation of the attention and visual processing of the children. A sequence of four A4-sized sheets of paper were presented to the children for 120 s per sheet. Each sheet was covered with very small pictures randomly printed across it. The children were asked to mark as many representations of the target picture—a bell, that was randomly interspersed among the other pictures—as quickly as they could. The total number of target pictures marked in the first 30 s of each of the four sheets (rapidity), and the total time (accuracy) provided the scores. Testing time: 10 min.

#### Appendix A.3.3. Short-Term Memory

##### Digit Span

Orsini A, Grossi D, Capitani E et al. Verbal and spatial immediate memory span: normative data from 1355 adults and 1112 children. Ital J Neurol Sci 1987; 6: 539–48.

Digit Span was used to assess the verbal memory of the children (Orsini A 1987). This test consisted of repetition of sequences of random numbers at the rate of one number per second. Beginning with two digits, if the children correctly repeated the required number sequence in three of five trials, the examiner increased the number of digits in the sequence. The test ended when the children failed to correctly repeat three trials. The number of elements the children recalled represented their memory span. Testing time: 5 min.

##### Corsi’s Block Tapping

Orsini A, Grossi D, Capitani E et al. Verbal and spatial immediate memory span: normative data from 1355 adults and 1112 children. Ital J Neurol Sci 1987; 6: 539–48.

Corsi’s Block Tapping was used to assess the spatial memory of the children (Orsini A 1987). Nine 1½-inch cubes were fastened to a board in a random order. The examiner tapped the blocks in a prearranged sequence and the children were asked to attempt to copy the tapping pattern. Here, the examiner determined the span for immediate recall of the children. By adding one tap to each successful sequence, the examiner determined the capacity of the children for immediate recall. Testing time: 5 min.

## Appendix B

**Table A1:** Raw scores in learning skills in third grade children.

Learning Skill	Sub-Skill	Raw Score (Mean ±SD)		
		Extremely Preterm	Very Preterm	Normative Sample
Reading	Text speed (Sill/sec)			
	*Entrance Test*	3.13 ± 0.46	2.85 ± 0.93	2.90 ± 1.10
	*Intermediate Test*	1.75	2.10 ± 1.41	2.99 ± 1.10
	*Final Test*	2.86 ± 0.17	2.34 ± 0.10	3.35 ± 1.10
	Text accuracy (Errors)			
	*Entrance Test*	1.00 ± 1.00	2.28 ± 2.21	4.90 ± 5.10
	*Intermediate Test*	4.00	2.17 ± 2.64	4.90 ± 5.00
	*Final Test*	4 ± 0.00	2.33 ± 3.21	4.10 ± 4.20
	Text comprehension (Correct answers)			
	*Entrance Test*	6.3 ± 2.08	8.5 ± 2.07	6.40 ± 2.70
	*Intermediate Test*	8.00	7.75 ± 2.06	7.30 ± 2.00
	*Final Test*	9.00 ± 0.00	9.33 ± 1.15	7.70 ± 4.80
	Word speed (Sill/s)	2.32 ± 0.71	2.13 ± 0.54	2.20 ± 0.70
	Word accuracy (Errors)	4.80 ± 3.27	3.90 ± 1.71	5.00 ± 4.00
	Non-word speed (Sill/sec)	1.42 ± 0.38	1.31 ± 0.34	1.40 ± 0.40
	Non-word accuracy (Errors)	8.67 ± 1.15	4.75 ± 3.20	6.00 ± 5.00
Spelling	Word accuracy (Errors)	3.66 ± 4.04	2.50 ± 1.24	2.00 ± 2.00
	Non-word accuracy (Errors)	3.00 ± 2.83	1.40 ± 1.14	4.00 ± 3.00
Mathematics	*Final Test*			
	AC-MT written calculation (Correct answers)	---	5.60 ± 2.07	6.51 ± 1.79
	AC-MT numerical knowledge (Correct answers)	---	20.80 ± 1.30	19.36 ± 4.00
	AC-MT accuracy (Errors)	---	3.80 ± 2.59	5.80 ± 5.60
	AC-MT speed (Sec)	---	124.00 ± 41.25	151.08 ± 90.33
	Battery for Developmental Dyscalculia	81.20 ± 17.20	88.50 ± 18.88	100.00 ± 15.00

**Table A2:** Raw scores in learning skills in fourth grade children.

Learning Skill	Sub-Skill	Raw Score (Mean ±SD)		
		Extremely Preterm	Very Preterm	Normative SAMPLE
Reading	Text speed (Sill/sec)			
	*Entrance Test*	3.07 ± 1.67	3.12 ± 0.88	2.50 ± 0.66
	*Final Test*	2.10 ± 0.00	3.43 ± 2.02	2.10 ± 0.00
	Text accuracy (Errors)			
	*Entrance Test*	3.12 ± 1.75	3.50 ± 2.54	5.00 ± 5.10
	*Final Test*	9.00 ± 0.00	2.75 ± 2.68	3.60 ± 4.00
	Text comprehension (Correct answers)			
	*Entrance Test*	7.00 ± 2.16	8.20 ± 1.14	7.20 ± 2.40
	*Final Test*	---	9.17 ± 1.17	8.40 ± 5.70
	Word speed (Sill/sec)	2.49 ± 1.40	2.60 ± 1.23	2.70 ± 0.70
	Word accuracy (Errors)	5.40 ± 5.41	3.36 ± 2.79	3.00 ± 3.00
	Non-word speed (Sill/sec)	1.27 ± 0.45	1.61 ± 0.45	1.70 ± 5.00
	Non-word accuracy (Errors)	10.40 ± 8.45	6.21 ± 4.65	5.00 ± 4.00
Spelling	Word accuracy (Errors)	3.00 ± 2.00	1.74 ± 2.07	1.00 ± 2.00
	Non-word accuracy (Errors)	4.00 ± 2.00	3.50 ± 2.87	3.00 ± 2.00
Mathematics	AC-MT written calculation (Correct answers)			
	*Entrance Test*	4.00 ± 4.24	5 ± 1.55	6.34 ± 1.60
	*Final Test*	---	7.00	6.62 ± 1.47
	AC-MT numerical knowledge (Correct answers)			
	*Entrance Test*	17.50 ± 3.53	17.33 ± 2.06	18.25 ± 3.44
	*Final Test*	---	18.00	18.09 ± 3.63
	AC-MT accuracy (Errors)			
	*Entrance Test*	13.00 ± 7.07	7.50 ± 3.45	6.38 ± 4.94
	*Final Test*	---	15.00	5.69 ± 4.43
	AC-MT speed (Sec)			
	*Entrance Test*	84	130.50 ± 38.10	117.54 ± 32.68
	*Final Test*	---	189.00	130.77 ± 53.52
	Battery for Developmental Dyscalculia	88	105.78 ± 12.97	100.00 ± 15.00

**Table A3:** Raw scores in learning skills in fifth grade children.

Learning Skill	Sub-Skill	Raw Score (Mean ±SD)		
		Extremely Preterm	Very Preterm	Normative Sample
Reading	Text speed (Sill/sec)			
	*Entrance Test*	2.43 ± 2.11	3.73 ± 1.11	3.77 ± 1.25
	*Final Test*	3.06 ± 1.55	3.20 ± 0.76	3.69 ± 1.12
	Text accuracy (Errors)			
	*Entrance Test*	5.00 ± 1.41	3.44 ± 2.88	5.9 ± 6.2
	*Final Test*	6.17 ± 5.74	5.57 ± 4.55	5.7 ± 5.9
	Text comprehension (Correct answers)			
	*Entrance Test*	9.00 ± 0.00	9.00 ± 1.51	7.6 ± 2.2
	*Final Test*	8.00 ± 1.09	6.14 ± 1.95	7.9 ± 1.8
	Word speed (Sill/sec)	3.13 ± 1.39	2.62 ± 1.31	3.20 ± 0.80
	Word accuracy (Errors)	3.22 ± 4.10	2.29 ± 2.33	3.00 ± 3.00
	Non-word speed (Sill/sec)	1.92 ± 1.00	1.78 ± 0.44	2.00 ± 0.60
	Non-word accuracy (Errors)	5.90 ± 7.92	3.09 ± 3.62	5.00 ± 4.00
Spelling	Word accuracy (Errors)	2.89 ± 3.29	2.66 ± 3.79	1.00 ± 1.00
	Non-word accuracy (Errors)	2.33 ± 1.15	3.10 ± 2.18	3.00 ± 3.00
Mathematics	*Entrance Test*			
	AC-MT written calculation (Correct answers)	6.42	7.40 ± 0.89	6.45 ± 1.49
	AC-MT numerical knowledge (Correct answers)	17.7	20.40 ± 2.07	18.38 ± 3.66
	AC-MT accuracy (Errors)	5.8	5.00 ± 5.66	5.75 ± 4.29
	AC-MT speed (Sec)	140	106.20 ± 43.37	120.7 ± 46.50
	Battery for Developmental Dyscalculia	85.33 ± 28.84	94.22 ± 23.63	100.00 ± 15.00

**Table A4:** Score in Neurocognitive skills.

Neurocognitive Skill	Sub-Skill		Mean ±SD		
			Extremely Preterm	Very Preterm	Normative Sample
Visual-motor integration (Standard Score)	Total	Years			
		8.00-8.11	93.20 ± 13.14	97.56 ± 13.52	100.00 ± 15.00
		9.00-9.11	100.75 ± 6.50	99.17 ± 14.62	100.00 ± 15.00
		10.00-10.11	101.30 ± 10.91	98.00 ± 11.79	100.00 ± 15.00
	Visual processing				
		8.00-8.11	110.17 ± 18.71	100.08 ± 17.19	100.00 ± 15.00
		9.00-9.11	92.00 ± 10.55	95.39 ± 15.80	100.00 ± 15.00
		10.00-10.11	104.00 ± 18.32	98.63 ± 15.06	100.00 ± 15.00
	Motor coordination				
		8.00-8.11	96.40 ± 5.41	105.45 ± 19.35	100.00 ± 15.00
		9.00-9.11	100.50 ± 17.60	101.26 ± 17.13	100.00 ± 15.00
		10.00-10.11	98.50 ± 14.45	104.00 ± 11.01	100.00 ± 15.00
Attention and visual processing (Raw score)	Selective				
		8.00-8.11	48.80 ± 11.77	49.57 ± 9.76	49.30 ± 9.80
		9.00-9.11	47.33 ± 14.29	54.95 ± 7.87	54.00 ± 10.00
		10.00-10.11	58.37 ± 10.36	59.42 ± 16.06	59.30 ± 14.70
	Sustained				
		8.00-8.11	116.00 ± 9.22	117.57 ± 10.11	119.20 ± 10.2
		9.00-9.11	117.66 ± 10.70	119.95 ± 1.07	124.30 ± 8.60
		10.00-10.11	121.75 ± 11.23	125.83 ± 10.82	128.10 ± 9.10
Short-term memory (Span)	Verbal				
		8.00-8.11	4.17 ± 0.75	4.33 ± 0.65	4.50 ± 1.10
		9.00-9.11	5.25 ± 1.26	4.91 ± 1.23	4.80 ± 1.10
		10.00-10.11	4.70 ± 0.67	4.88 ± 0.88	5.40 ± 2.10
	Visuospatial				
		8.00-8.11	4.00 ± 0.00	4.00 ± 0.43	4.30 ± 0.90
		9.00-9.11	4.50 ± 1.00	4.60 ± 1.14	4.60 ± 0.80
		10.00-10.11	4.80 ± 0.79	5.31 ± 0.95	4.80 ± 0.80
